# Associations between kindergarten climate and retention intention of kindergarten teachers: The chain mediating roles of perceived organizational support and psychological empowerment

**DOI:** 10.3389/fpsyg.2022.906434

**Published:** 2022-08-01

**Authors:** Dasheng Shi, Mengmeng Zhang, Yan Wang, Yongqi Xu, Xiantong Yang

**Affiliations:** ^1^School of Education, Minzu University of China, Beijing, China; ^2^School of Psychology, Beijing Normal University, Beijing, China

**Keywords:** kindergarten climate, perceived organizational support, psychological empowerment, retention intention, kindergarten teacher

## Abstract

Kindergarten climate has been reported to be closely associated with teachers' retention intention, yet the underlying mechanism of this association remains unclear in some ethnic minority areas in China. Based on the Personal-Environment Fit Theory and Organizational Support Theory, the research aims to examine the correlation between kindergarten climate and retention intention of Chinese kindergarten teachers in ethnic minority areas and the chain mediating role of perceived organizational support, as well as the psychological empowerment. In total, 1,199 Chinese kindergarten teachers were recruited from ethnic minority areas to complete the questionnaires. Based on their responses, the main findings of the study were listed as follows: (1) A supportive kindergarten climate has a positive correlation with perceived organizational support, psychological empowerment, and retention intention. (2) A positive kindergarten climate would increase the retention intention only through the indirect path of perceived organizational support, psychological empowerment, as well as the chain mediating path between these two variables. Taken together, these results further explained the interplay between kindergarten climate and teachers' retention intention. Implications for research on retention intention were discussed. Theoretically, it enriches the theoretical basis related to the external environmental resources and individual cognition. Practically, it means that educational policymakers will need to issue new guidelines to ensure that more kindergarten teachers are retained in China's ethnic minority areas.

## Introduction

This issue of teacher attrition has been getting greater attention in China's ethnic minority areas (Yu and Zhang, 2018), especially for kindergarten teachers in these areas. The attrition of kindergarten teachers in ethnic minority areas would lower the local educational quality (Long and Yuan, 2021) and therefore waste public resources invested in teacher teaching (Van den Borre et al., 2021). To some extent, it would also jeopardize the morale of the remaining teacher population in ethnic minorities (Zhang and Yang, 2018) and decrease the overall quality of education in China (Pang, 2019). Therefore, it is vital to investigate kindergarten teacher retention in ethnic minority areas.

The rising teacher turnover rate may attribute to the declining attractiveness of teacher career (e.g., high workload, low salary, and poor working conditions; Long and Yuan, 2021). According to a survey of kindergarten teachers in China, 38.8% of kindergarten teachers intend to change their current kindergarten job (Hong et al., 2021). In general, one's magnitude of turnover intention would be the stellar opposite to one's retention intention, which refers to the intent that an individual stays in their current post. It is a reflection of an individual's self-reflection of the teacher willingness to continue working in the organization (Price and Mueller, 1981). Previous studies further investigated the correlation between teachers' work environment, individual's psychological functioning, and their retention in separate studies (Lee and Nie, 2014; Cheng et al., 2020; Ji and Yue, 2020). However, there is a void of academic studies that examined the issue from a comprehensive perspective that integrated external environment and internal psychology beliefs. According to the research, kindergarten climate belongs to the external environment factor and emerges as a vital antecedent factor affecting employee retention intention (Li et al., 2016; Lan et al., 2020). Organizational support perceived by employees and psychological empowerment are important belief factors that affect teachers' intention to retention (Armstrong-Stassen and Ursel, 2009; Ma et al., 2021). The mechanism of how external environmental factors and psychological belief factors affect teacher retention intention has not been confirmed. Notably, most of the previous studies only investigated the retention intention of employees and novice teachers (Fletcher et al., 2018; Keese et al., 2022), but failed to include any discussion on related issues in the kindergarten stage, especially in the context of ethnic minority areas in China.

Our study first aims to explore the influencing factors of kindergarten teachers' retention intention in ethnic minority areas based on personal-environment fit theory and organizational support theory, which help to deepen our understanding about how to retain teachers and provide intervention schemes for reducing the turnover rate. Second, to further unravel the underlying mechanism of teachers' retention intention in China's minority areas, we aim to examine the mediators (i.e., perceived organizational support and psychological empowerment) between kindergarten climate and retention intention, thereby providing effective suggestions for promoting the retention of kindergarten teachers from the perspective of management psychology.

### Kindergarten climate and retention intention

In a kindergarten setting, kindergarten climate may be one of the important factors affecting teachers' intention to stay in school (Thapa et al., 2013; Hatziconstantis and Kolympari, 2021). The term “kindergarten climate” refers to all aspects of the kindergarten environment that organizational members consciously perceive, as well as the quality and characteristics of a kindergarten (Collie et al., 2012). Based on the definition of Liu (2020), it manifested itself in five specific constructs, including physical climate, learning climate, interpersonal climate, institutional climate, and external climate. Based on the personal-environment fit theory (Kristof-Brown et al., 2005), when the external work climate meets employees' basic psychological needs, employees are motivated to contribute to the organization's growth and demonstrate positive behaviors (Thapa et al., 2013; Berberoglu, 2018). In other worker populations, such as pharmacy workers, it has been proven that a negative work climate will increase the likelihood of pharmacists to leave their current jobs (Lan et al., 2020). On the contrary, previous research indicates that a favorable work climate, particularly a benign interpersonal climate (characterized by harmonious relationships with colleagues and leadership), is frequently cited as a key reason for teachers to remain with the same organization (Liu, 2012). By contrast, when teachers perceived a negative kindergarten climate, such as the lack of developmental opportunities, facing conflicts and contradictory colleague relationships, teachers' sense of stress will increase, resulting in burnout or resignation finally (Liu, 2012; Shorosh and Berkovich, 2022). Therefore, kindergarten climate may be closely related to the retention intention of kindergarten teachers.

### Kindergarten climate, organizational support, and retention intention

Perceived organizational support may be a significant mediating variable in the association between kindergarten climate and retention intention. Perceived organizational support refers to the organization's attention to employees' contributions and concern for their wellbeing (Eisenberger et al., 1986). Based on the organizational support theory, organizational member's perception of positive organizational climate contributes to the formation of positive work beliefs and motivation. Previous studies indicated that employees working in a positive interpersonal climate (e.g., organization caring for employees and their families) and a just institutional climate (e.g., organization implements procedural justice work content) are more likely to perceive organizational support (Zhang et al., 2012, 2017). A meta-analysis related to perceived organizational support also identified that institutional justice in the work climate is an important factor influencing workers' perception of organizational support (Rhoades and Eisenberger, 2002). Additionally, interpersonal climate and institutional climate are all critical components of the organizational climate. In other words, organizational climate is strongly correlated with perceived organizational support (Köse and Campus, 2016).

Furthermore, perceived organizational support may exert potential effects on retention intention. Perceived organizational support is generally assumed to reflect employees' norms and values and to influence their behavior tendency (Li et al., 2020). If teachers are aware that their organization values their opinions and voices, they will be motivated to work and care about the kindergarten's development (Yang et al., 2018; Matsuo et al., 2021). As a result, they may not seek other work opportunities and stay in the present position. Empirical studies indicated that perceived organizational support could reinforce retention intention among employees (Armstrong-Stassen and Ursel, 2009; Feng and Angeline, 2010; Li et al., 2020). In summary, we could reasonably propose that kindergarten climate was a facilitator of perceived organizational support, which would in turn improve the retention intention among Chinese ethnic minority kindergarten teachers.

### Kindergarten climate, psychological empowerment, and retention intention

Psychological empowerment may be a vital mediator between kindergarten climate and retention intention. Psychological empowerment is defined as the synthesis of the psychological state or cognition experienced by an individual, which is manifested in four aspects, namely, meaning, competence, self-determination, and impact (Thomas and Velthouse, 1990). Meaning refers to the value judgment made by individuals on current tasks and goals based on their own values and standards. Competence refers to the degree to which an individual perceives his or her ability to complete a task. Self-determination is individuals' awareness that they have autonomy in how they regulate their behavior. Impact refers to the individuals' awareness that they have an important influence on the organizational environment (Thomas and Velthouse, 1990). Theory of empowerment holds that individual psychological empowerment is influenced by the organizational climate (Conger and Kanungo, 1988). According to the opinion of Wang et al. (2013), organizational climate that provides supportive material and psychological resources, as well as career opportunities, is more likely to empower employees and enable them to successfully complete their work. Another study found that a supportive work climate contributes to employees' participation in organizational decision-making, showing higher psychological empowerment (Carless, 2003). Therefore, the work climate could positively affect psychological empowerment.

Furthermore, psychological empowerment also reflects an individual's active rather than passive orientation toward work roles (Zimmerman, 1995). Individuals with greater empowerment will experience the meaning and competence of their work and value the sense of autonomy (Ma et al., 2021). These positive experiences and feelings altogether would reinforce their sense of approval for the job and then the willingness to stay in the same organization. Similarly, some empirical studies also indicated that psychological empowerment has a positive association with retention intention (Erturk and Vurgun, 2015; Ma and Zhou, 2021; Ma et al., 2021). Overall, we could reasonably hypothesize that a supportive kindergarten climate may be a positive predictor of psychological empowerment, which in turn may improve the retention intention among Chinese ethnic minority kindergarten teachers.

### Perceived organizational support and psychological empowerment

Perceived organizational support may predict psychological empowerment. According to social exchange theory, individuals will be motivated to help the organization thrive when they perceive organizational care and attention. As Özaralli (2003) indicated, employees who perceive organizational support in the workplace believe they are better competent in completing tasks. Competence is a manifestation of positive psychological empowerment (Thomas and Velthouse, 1990). Empirical research found that perceived organizational support could positively influence psychological empowerment (Ertürk, 2010; Caesens et al., 2020). Likewise, empowered individuals who received more support from the organization considered themselves as an important part of the organization, experiencing a sense of control over their work and actively participating in organizational activities (Chiang and Hsieh, 2012). The abovementioned studies bolstered the hypothesis by demonstrating that perceived organizational support was positively related to psychological empowerment. In addition, a positive kindergarten climate may satisfy the psychological needs of employees and make them perceive the organization's support, resulting in strong psychological empowerment and a greater intention to maintain with the same organization. Therefore, based on the above review, we could plausibly predict that the influence of kindergarten climate on retention intention of Chinese kindergarten teachers may be mediated by perceived organizational support and psychological empowerment.

### The present study

**Figure 1:**
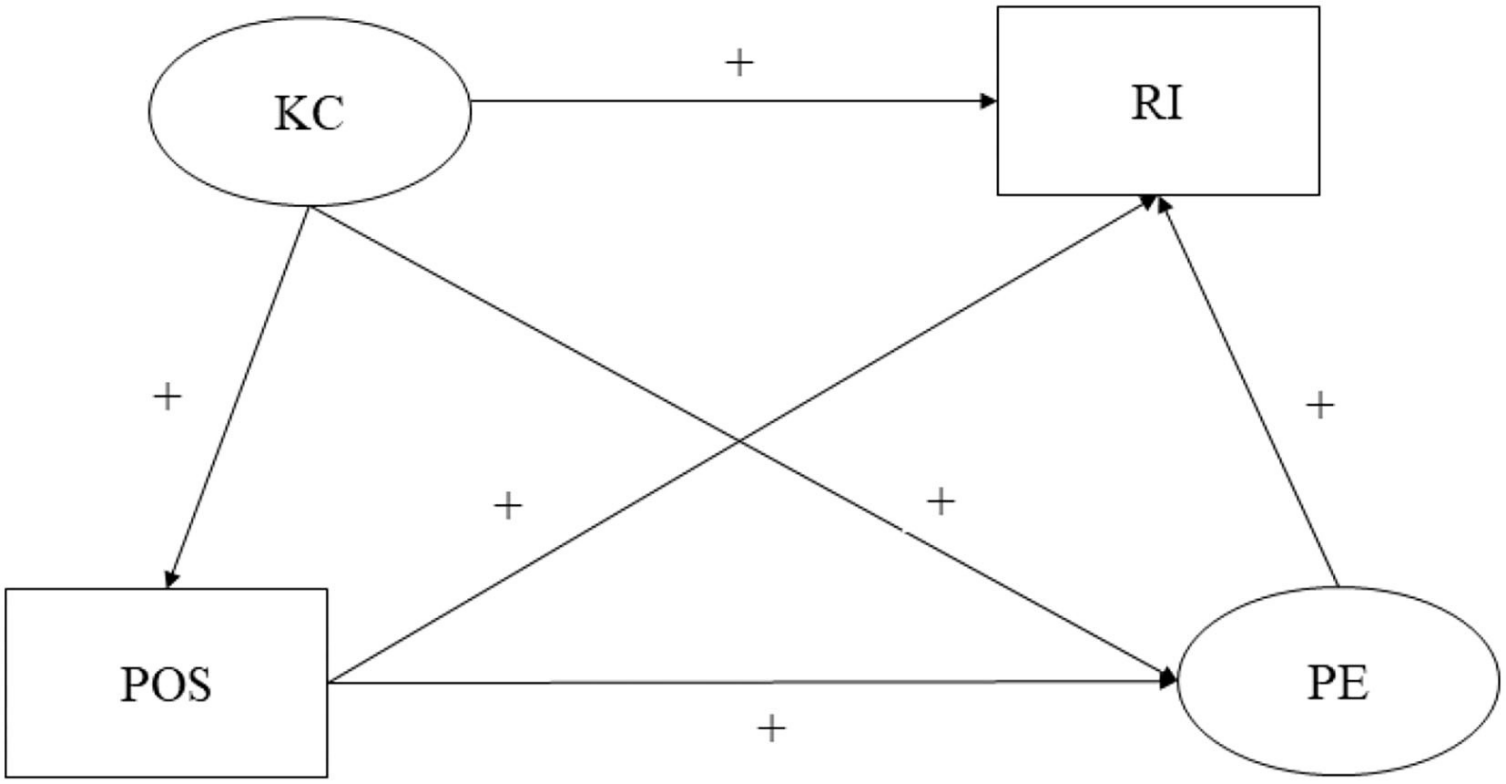
Conceptual framework of organizational support and psychological empowerment as mediators. KC, kindergarten climate; RI, retention intention; POS, perceived organizational support; PE, psychological empowerment.

Based on previous research findings on kindergarten climate and retention intention, the study aims to investigate whether perceived organizational support and psychological empowerment would mediate the association between kindergarten climate and retention intention of kindergarten teachers in ethnic minority areas of China. Based on several theoretical frameworks, an integrated hypothesized model (Figure 1) was proposed to reveal the complex association. This study proposed the hypothesis that a supportive kindergarten climate would have a positive impact on the retention intention of kindergarten teachers in ethnic minority areas of China (H1). Perceived organizational support would play mediating role in the association between kindergarten climate and retention intention among Chinese ethnic minority kindergarten teachers (H2). That is, supportive kindergarten climate may be a positive predictor of perceived organizational support, which in turn may improve the retention intention among Chinese ethnic minority kindergarten teachers. Psychological empowerment would play mediating role in the association between kindergarten climate and retention intention among Chinese ethnic minority kindergarten teachers (H3). That is, supportive kindergarten climate may be a positive predictor of psychological empowerment, which in turn may improve the retention intention among Chinese ethnic minority kindergarten teachers. Moreover, the kindergarten climate would exert effects on Chinese kindergarten teachers' retention intention through the chain mediating of perceived organizational support and psychological empowerment (H4). That is, supportive kindergarten climate may be a positive predictor of perceived organizational support, and perceived organizational support could positively predict psychological empowerment, which in turn may improve the retention intention among Chinese ethnic minority kindergarten teachers.

## Methods

### Participants

In this study, we adopted a convenient sampling method to collect teachers' data from 32 kindergartens in Ningxia Hui Autonomous Region, Haixi Mongolian and Tibetan Autonomous Prefecture, Kunming, Yunnan Province from 1 to 20 November 2021 using the online questionnaire platform. To ensure the questionnaire quality, we first contacted the principal of the kindergarten to explain our research intention. The questionnaire was delivered with the approval of the principal. To guarantee that teachers could fill out the questionnaire according to their true desires, we first told teachers to participate in the study project voluntarily. Second, teachers who took part in the project completed the questionnaire in a relaxed and undisturbed setting. Finally, the questionnaires were filled out anonymously to minimize the impact of outside pressure on the participants' responses; 1,256 questionnaires were retained for teachers and 1,199 were valid (for detailed participants' characteristics, see Supplementary material). Among the participants, 1,159 were female respondents (96.7%) and only 40 were male respondents (3.30%); 878 respondents (73.2%) had been teaching for ≤2 years; 321 respondents (26.8%) had taught for >2 years; 283 respondents (23.6%) had a high school education; 911 respondents (76%) had a bachelor's degree; and five teachers (0.4%) had a master's degree or higher. Nearly 65% of teachers (768) are Han, 33.5% of teachers (402) are Hui, and the remaining samples came from various other ethnic groups (e.g., Bai, Tibetan, Dai, Lisu, Man, Miao, and Yi) in China. All measurements and procedures were permitted by the Institutional Review Board (IRB) of the first author's institution.

### Measures

The questionnaire items in this study were translated and adapted from prior studies. To verify the validity of the instrument's content, we invited three experts from educational psychologists to review it. These questionnaires were first sent to kindergarten teachers with similar work experience to the target participants for preliminary testing. Some items were modified correspondingly to ensure face validity. The instrument was evaluated using a 5-point Likert scale ranging from 1 (*strongly disagree*) to 5 (*strongly agree*).

#### Kindergarten climate

The kindergarten climate assessment scale prepared by the “Social Emotional Learning” project organized by the Ministry of Education of China and UNICEF was used to measure the kindergarten climate (Liu, 2020). This survey was adapted for the research questions to include four kindergarten climate dimensions, namely, physical climate, learning climate, interpersonal climate, and institutional climate. Physical climate inquired about the hardware facilities and environment settings (e.g., “To what extent do you think the kindergarten environment is clean and tidy?”). Learning climate asked whether kindergartens provide learning and development support for teachers (e.g., “To what extent do you think kindergartens provide teachers with opportunities to improve their professional knowledge and skills?”). Interpersonal climate refers to teachers' collaborations with their colleagues (e.g., “To what extent do you think you get along very well with colleagues?”). Institutional climate reflects the sense of teacher's identity and belonging of the kindergarten, and the degree of participation in the management and construction of the kindergarten (e.g., “To what extent do you think the kindergarten rules adequately consider the needs of teachers?”). Items are assessed on a 5-point Likert scale ranging from one (*completely disagree*) to five (*completely agree*). Previous research also verified that this scale had a good internal consistency (Cronbach's α = 0.917; He et al., 2021). The Cronbach's alpha for this scale was 0.975, and the Cronbach's alpha of sub-dimensions ranges from 0.940 to 0.962, revealing a good internal consistency. The confirmatory factor analysis showed a good fit [χ^2^*/df* = 9.42, *p* < 0.001, RMSEA (90% CI) = 0.084 (0.077, 0.091), CFI = 0.980, TLI = 0.971, SRMR = 0.021].

#### Retention intention

The kindergarten teacher retention intention scale prepared by Guo and Sun (2018) was adopted, which contains four items (e.g., “I plan to continue working as a kindergarten teacher.”). Respondents rated on a 5-point Likert scale, ranging from one (*completely disagree*) to five (*completely agree*), with higher scores demonstrating higher retention intention. Additionally, Guo and Sun (2018) evaluated pre-service kindergarten teachers' retention intention and validated the scale's internal consistency (Cronbach's α = 0.92). The Cronbach's alpha for this scale in our study was 0.910. The confirmatory factor analysis showed a good fit [χ^2^*/df* = 2.9, *p* < 0.001, RMSEA (90% CI) = 0.040 (0.000, 0.079), CFI = 0.998, TLI = 0.995, SRMR = 0.009].

#### Perceived organizational support

The perceived organizational support scale created by Hekman et al. (2009) with eight questions was used to assess perceived organizational support (e.g., “My organization cares about how satisfied I am with my job”). A 5-point rating was used, which ranges from one “*strongly disagree*” to five “*strongly agree*,” with high scores indicating a high degree of organizational support. Shen and Benson (2016) guided a scale survey among company employees and confirmed a good internal consistency (Cronbach's α = 0.83). The Cronbach's alpha for this scale in our study was 0.775. The confirmatory factor analysis showed a good fit [χ^2^*/df* = 5.29, *p* < 0.001, RMSEA (90% CI) = 0.060 (0.048, 0.073), CFI = 0.988, TLI = 0.979, SRMR = 0.032].

#### Psychological empowerment

Psychological empowerment was measured using the psychological empowerment scale (Spreitzer, 1995), which had been revised and validated in Chinese and demonstrated acceptable reliability and validity (Li et al., 2006). The scale consists of 12 items in four dimensions, namely, meaning (three items, e.g., “The work I do is very meaningful to me”), self-determination (three items, e.g., “I can decide how to do my work”), self-efficacy/competence (three items, e.g., “I have confidence in my ability to do the job”), and impact (three items, e.g., “I have significant influence in my work”). It was rated on a 5-point scale, ranging from one (*strongly disagree*) to five (*strongly agree*), with higher scores representing higher psychological empowerment. Previous studies also indicated that this scale had good internal consistency (Cronbach's α > 0.8) in China (Wang and Pan, 2014; Kong and Qian, 2015). This study revealed a satisfactory internal consistency in total scale (Cronbach's α = 0.943) and sub-dimensions (Cronbach's α = 0.868–0.922). The confirmatory factor analysis showed a good fit [χ^2^*/df* = 6.03, *p* < 0.001, RMSEA (90% CI) = 0.065 (0.057, 0.073), CFI = 0.984, TLI = 0.977, SRMR = 0.025].

## Results

In this study, descriptive analyses and Pearson's correlations were first performed using SPSS 23.0 version to estimate the means, standardized deviations, and correlation between the major variables. Second, the structural equation model (SEM) was used to analyze the mediating effects of organizational support and psychological empowerment on kindergarten climate and retention intention by using Mplus 7.1 version, after controlling gender and seniority. Besides, we use maximum likelihood (ML) to handle the missing data in the statistical analyses, and the following fitting statistical scores were calculated to determine the degree of fit between the survey data and the hypothesis model: Chi-square values (χ^2^) test of difference, the comparative fit index (CFI), and Tucker-Lewis fit index (TLI) with a value more than 0.90, the root mean squared error of approximation (RMSEA) with a value <0.08, and the standard root mean squared residual (SRMR) with a value near 0.05 are all indicators of a good fit (Wen et al., 2004).

### Common method variance analysis

Since the data rely on kindergarten teachers' subjective self-reports, there may be some covariations, which means that common method bias needs to be examined. The Harman single factor test was used to determine the common method deviation or systematic measurement error (Harman, 1976). Six factors had eigenvalues greater than one, as shown by the findings. The first factor explained 38.57% of total variation, less than the 40% threshold criterion (Podsakoff et al., 2003), indicating that no significant common method bias existed.

### Descriptive and correlation analyses

The descriptive analysis and correlation analysis of the major variables are provided in Table 1. The results showed significant correlations between age and perceived organizational support, retention intention, psychological empowerment, and seniority; and perceived organizational support and seniority had significant correlations, which suggested that subsequent analyses need to consider age and seniority as control variables. Additionally, there was a positive correlation between kindergarten climate, perceived organizational support, retention intention and psychological empowerment (*r* ranged from 0.404 to 0.752).

**Table 1:** Means, standard deviations, and correlations of the variables (*N* = 1,199).

**Variables**	***M***	***SD***	**1**	**2**	**3**	**4**	**5**	**6**	**7**
1. Kindergarten climate	3.988	0.955	1						
2. Perceived organizational support	3.385	0.666	0.404^*^	1					
3. Retention intention	3.803	0.919	0.487^*^	0.453^*^	1				
4. Psychological empowerment	3.798	0.698	0.517^***^	0.439^*^	0.752^***^	1			
5. Age	3.319	1.664	0.031	0.161^*^	0.093^*^	0.082^*^	1		
6. Gender	1.970	0.180	−0.018	−0.034	0.004	−0.008	−0.082^*^	1	
7. Seniority	1.267	0.443	−0.017	0.079^*^	0.030	0.052	0.553^*^	−0.087^*^	1

*** *p < 0.001*,

** *p < 0.01*.

*All tests were two-tailed*.

### Chain mediation model analysis

This study used a mediation model to explore the effects of perceived organizational support and psychological empowerment on kindergarten climate and retention intention. Based on SEM approach (Cheung, 2007), the chain mediation model indicated a satisfactory model fit (χ^2^*/df* = 5.9, RMSEA = 0.064, CFI = 0.945, SRMR = 0.057). To examine the mediating impact, the non-parametric percentile bootstrap approach with bias correction was utilized. For the analysis, the bootstrap approach with 1,000 bootstrap samples was adopted. Table 2 revealed the SEM path coefficients. The results showed that kindergarten climate had a significant predictive effect on psychological empowerment (β = 0.463, *p* < 0.001) and perceived organizational support (β = 0.426, *p* < 0.001), but did not significantly predict retention intention (β = 0.016, *p* > 0.05). Perceived organizational support significantly predicted psychological empowerment (β = 0.290, *p* < 0.001) and retention intention (β = 0.057, *p* < 0.01). Psychological empowerment significantly predicted retention intention (β = 0.796, *p* < 0.001).

**Table 2:** SEM path coefficients.

**SEM path**	**Standardized**		**Non-standardized**	
	***β***	***SE***	***β***	***SE***
Kindergarten climate to psychological empowerment	0.463^***^	0.039	0.351^***^	0.038
Perceived organizational support to psychological empowerment	0.290^***^	0.033	0.304^***^	0.304
Kindergarten climate to perceived organizational support	0.426^***^	0.029	0.309^***^	0.209
Psychological empowerment to retention intention	0.796^***^	0.025	1.052^***^	0.032
Kindergarten climate to retention intention	0.016	0.023	0.016	0.023
Perceived organizational support to retention intention	0.057^**^	0.023	0.079^*^	0.031

*All SE are jackknifed standard errors*.

*** *p < 0.001*,

** *p < 0.01*,

* *p < 0.05*.

**Figure 2:**
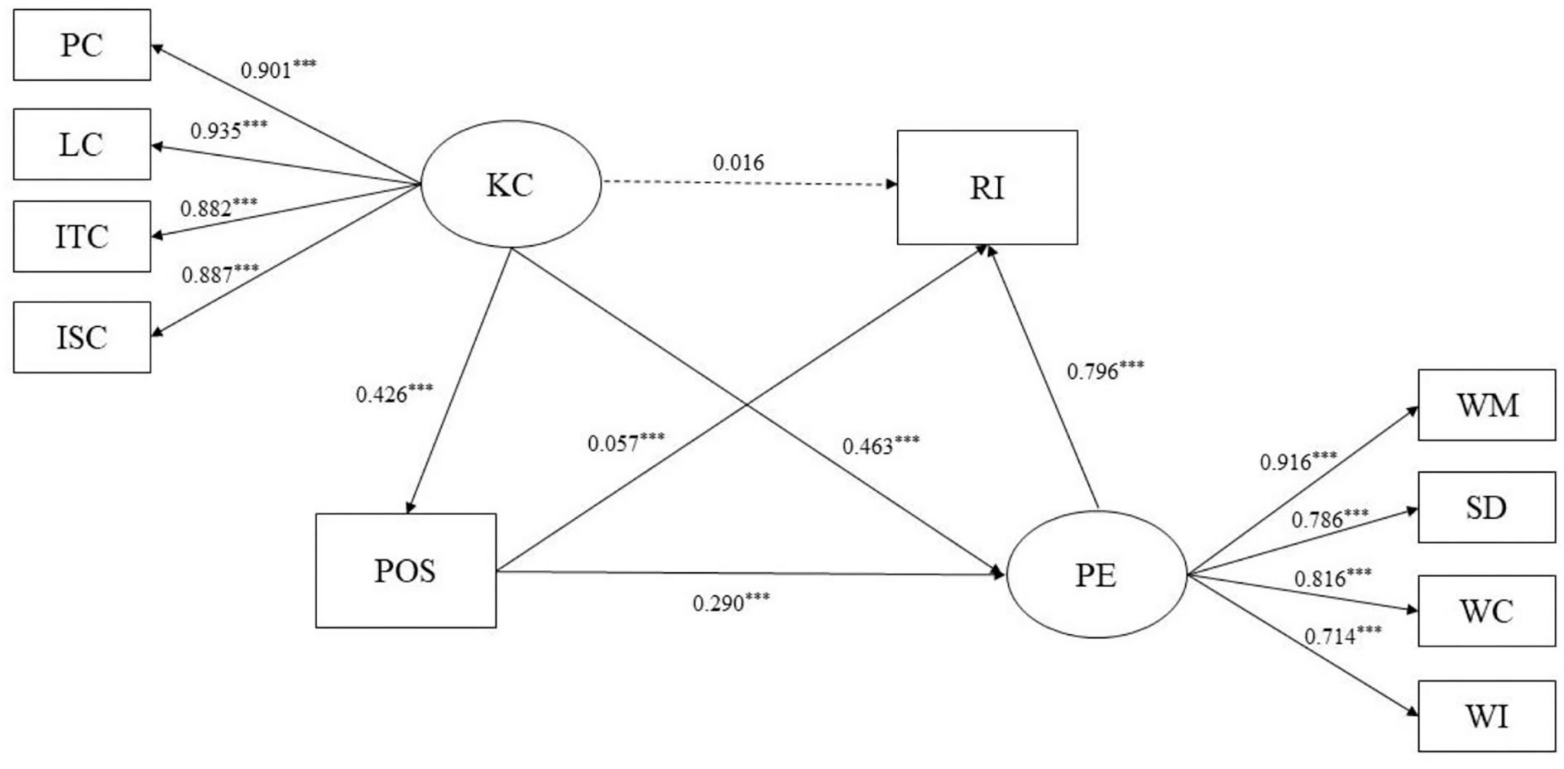
The chain mediation model. ***p < 0.001. KC, kindergarten climate; RI, retention intention; POS, perceived organizational support; PE, psychological empowerment; PC, physical climate; LC, learning climate; ITC, interpersonal climate; ISC, institutional climate; WM, work meaning; SD, self-determination; WC, work competence; WI, work impact.

Table 3 showed the mediating effects of perceived organizational support and psychological empowerment between kindergarten climate and retention intention. Figure 2 was a chain mediating model. The direct effect of teachers' perceived kindergarten climate on retention intention was 0.016 [95% CI: (−0.027, 0.066)], indicating that the direct effect could not be confirmed. To further verify the mediating effect of perceived organizational support and psychological empowerment on kindergarten climate and retention intention, the results ranged from 0.076 to 0.126 (95% CI, not including zero), demonstrating that perceived organizational support and psychological empowerment mediated the relation between kindergarten climate and retention intention, and the Total standardized Mediating effect value was 0.491 [95% CI: (0.428, 0.603)]. In particular, the mediating effect comprised three indirect effects, namely, path 1: kindergarten climate → perceived organizational support → retention intention (0.024); path 2: kindergarten climate → psychological empowerment → retention intention (0.369); and path 3: kindergarten climate → perceived organizational support → psychological empowerment → retention intention (0.098). The ratios of the three indirect effects to the total effect were 4.73, 72.78, and 19.33% for paths 1, 2, and 3, respectively. Hypotheses 2, 3, and 4 could be confirmed.

**Table 3:** Perceived organizational support and psychological empowerment in the mediating effect analysis.

**Effect**	**Path relationship**	**Effect size**	**Bootstrap 95% CI**	**Relative mediation effect**
Direct effect	kindergarten climate → retention intention	0.016	[−0.027, 0.066]	3.16%
Path 1	kindergarten climate → perceived organizational support → retention intention	0.024	[0.007, 0.047]	4.73%
Path 2	kindergarten climate → psychological empowerment → retention intention	0.369	[0.291, 0.456]	72.78%
Path 3	kindergarten climate → perceived organizational support → psychological empowerment → retention intention	0.098	[0.076, 0.126]	19.33%
Total mediating effect		0.491	[0.428, 0.603]	96.84%
Total effect		0.507		100%
Compare 1		−0.345	[−0.433, −0.267]	
Compare 2		−0.074	[−0.109, −0.043]	
Compare 3		0.271	[0.189, 0.362]	

Furthermore, we compared the indirect effects of different paths in pairs to examine if there were significant path differences. In comparison 1, the bootstrap 95% confidence interval for the difference between indirect effects 1 and 2 did not include 0 [95% CI: (−0.433, −0.267)], representing a statistically significant difference between the two. By the same method of comparison, there were significant difference between indirect effects 1 and 3 [comparison 2, 95% CI: (−0.109, −0.043)] or between indirect effects 2 and 3 [comparison 3, 95% CI: (0.189, 0.362)].

## Discussion

Our research contributes to the existing literature by investigating the underlying mechanisms between kindergarten climate and retention intention with a sample of kindergarten teachers in ethnic minority areas of China. In this study, we tested a chain mediation model in which perceived organizational support mediated the association between kindergarten climate and retention intention; psychological empowerment mediated the association between kindergarten climate and retention intention; and kindergarten climate might indirectly influence teachers' retention intention through the chain mediating effect of perceived organizational support and psychological empowerment. Taken together, these pathways provide a comprehensive and constructive viewpoint that sheds light on the link between kindergarten teachers and retention intention.

### The association between kindergarten climate and retention intention

As the results showed, after introducing two mediating variables of perceived organizational support and psychological empowerment, we found that kindergarten climate is unrelated to kindergarten teachers' intention to remain in ethnic minority areas, but regression analysis found that kindergarten climate could exert effects on the retention intention (β = 0.466, *p* < 0.001; see Supplementary material). This suggested that the effect of kindergarten climate on retention intention was influenced indirectly by perceived organizational support and psychological empowerment rather than directly by the amount of external environmental resources. To some extent, this finding was inconsistent with prior studies (Liu, 2012; Lan et al., 2020) and refuted the view that environmental climate would directly affect individual behavior. More specifically, it indicated that the impact of kindergarten climate on teachers' retention intention is not direct and obvious; rather, it is a complex mechanism that takes various psychological variables into consideration.

### The mediating role of perceived organizational support

According to the results of the mediation analysis, perceived organizational support mediated the association between kindergarten climate and retention intention. This was consistent with our hypothesis 2. In terms of the first stage of the mediation link (e.g., from kindergarten climate to perceived organizational support), kindergarten climate is positively associated with perceived organizational support, which supported the organizational support theory (Eisenberger et al., 1986). Perceived organizational support explained whether or not an organization appreciates and cares about its employees, as well as how supportive it is of its employees (Appelbaum et al., 2019). In this case, the magnitude of teachers' perception toward organizational support is determined by the organizational climate. If the organizational climate is a cooperative, sharing, and supportive one, teachers' perception of organizational support will be high, whereas, if teachers are in a lack of cooperation or a restrictive school climate, it is difficult for teachers to perceive organizational support for personal development (Silva et al., 2017). Besides, the results also demonstrated that perceived organizational support had a potential predictive effect on the retention intention of kindergarten teachers, which is in line with the previous research findings (Armstrong-Stassen and Ursel, 2009; Boakye et al., 2021). A higher individual's retention tendency reflects an individual's positive self-perception to an organization (Price and Mueller, 1981). When employees perceive the organization's support and feel accepted by the organization, they are motivated to work and care about the development of the organization (Li et al., 2020). Taken together, our findings showed that in a positive and shared kindergarten climate, teachers perceived material and spiritual support from the organization, which can effectively improve employees' positive experience of their work and show their willingness to stay in the kindergarten in ethnic minority areas.

### The mediating role of psychological empowerment

Regarding psychological empowerment, the findings of this study showed that the kindergarten climate could promote retention intention by enhancing psychological empowerment, which was consistent with our hypothesis 3 and supported empowerment theory (Conger and Kanungo, 1988). Regarding the first step of the mediation connection (e.g., from kindergarten climate to psychological empowerment), the kindergarten climate is positively associated with psychological empowerment. Empowerment is an intrinsic psychological process that occurs when a person feels motivated in the workplace. For example, organizational environments that provide supportive material and psychological resources, as well as promotion opportunities, are more likely to empower employees and enable them to successfully accomplish their work (Wang et al., 2013). Furthermore, psychological empowerment predicted retention intention, confirming the theory of psychological empowerment. The core belief of teachers' work cognition is the individual's subjective feeling of work experience. Effective empowerment enabled teachers to behave properly based on actionable and articulated goals, believe that they are competent for the work, and experience the ability to control the work (Ma et al., 2021), which would effectively improve the psychological state of kindergarten teachers, enhancing their work motivation and intention to stay in the kindergarten in ethnic minorities. Taken together, our findings indicated that a supportive work climate reinforced teachers' perception of empowerment, and in turn, kindergarten teachers are more likely to be motivated to engage in teaching and show stronger intention to stay in the same organization.

### The chain mediating role of perceived organizational support and psychological empowerment

Finally, it was found that the kindergarten climate could enhance the retention intention of kindergarten teachers in ethnic minority areas of China by promoting perceived organizational support and psychological empowerment, which was consistent with our hypothesis 4 and verified personal-environment fit theory (Kristof-Brown et al., 2005), organizational support theory (Eisenberger et al., 1986), psychological empowerment theory (Conger and Kanungo, 1988), and social exchange theory (Cropanzano and Mitchell, 2005). The finding indicated that the influence process of kindergarten climate on retention intention was relatively complex. To be specific, when ethnic minority kindergarten provides a positive climate, teachers in this context are more likely to experience stronger organizational support and to feel that they have enough resources to perform work efficiently. In this way, they would further hold higher levels of psychological empowerment and intend to stay in ethnic areas to teach. This study extends the literature by providing empirical evidence that kindergarten can retain teachers by building supportive work climate (e.g., interpersonal support and resource support) and empowering them with decision-making power, control power, and so on.

## Conclusion and recommendations

### Limitations and future study

There are several limitations to this study. First, cross-sectional research could not establish causal linkages across models; longitudinal and experimental investigations are needed to confirm these associations. Second, this study treated kindergarten climate and psychological empowerment as a whole variable, without distinguishing the influence of specific dimensions on retention intention. Future research should further explore whether there are differences in the internal mechanisms of the influence of specific dimensions on retention intention. Third, there could be sample bias when collecting only self-reported data so that the validity of the research could be diminished. Therefore, multiple measures are required to reduce biases and enhance reliability, such as third-party observation. Finally, most of our study participants are female kindergarten teachers. Despite our attempts to establish a balanced gender ratio of research participants, field studies have indicated that the number of male kindergarten teachers is relatively low. Future research should adopt qualitative methods to explore the reasons why male kindergarten teachers teach in minority areas.

### Implications

This study has several theoretical and practical implications. To our knowledge, this is the first study to investigate the potential chain mediating effect of kindergarten climate on teachers' retention intention in ethnic minority areas of China. From a theoretical perspective, it is beneficial to investigate the process mechanism of kindergarten climate influencing Chinese kindergarten teachers' retention intention, which may provide a better understanding of the antecedents that impact retention intention and enrich the theoretical basis related to the external environmental resources and individual cognition.

From a practical perspective, our study has important implications for educational decision-makers who aim at facilitating kindergarten teacher's retention in ethnic minority areas in China. This research results highlighted that kindergarten climate could affect the teachers' retention intention through perceived organizational support, psychological empowerment, and the chain mediating effect between the two. This is noteworthy, especially in the context of the Chinese ethnic minority areas, where perceived organizational support and psychological empowerment are major predictors of kindergarten teachers' retention intention. On the one hand, teachers' needs should be satisfied to improve their sense of organizational support. Local education administrators and policymakers should strive to understand that every teacher has different necessities and expectations and address individual teacher's necessities. If a teacher received the support, they would perceive it as a favorable characteristic of a kindergarten and then likely to be retained in the same kindergarten. For example, the welfare benefits of kindergarten teachers in ethnic minority rural areas are lower than those of urban teachers, which leads to a greater sense of psychological gap inevitably. Thus, kindergarten and local authorities should provide more support to these teachers, including motivating and harmonious working climate, flexible working schedule, and competitive salary in the local areas, thereby alleviating their sense of comfortlessness. Only when they are financially supported and granted with respect to seek self-esteem, teachers' intention to stay on will be improved.

On the other hand, kindergarten administrator should pay attention to the construction of psychological empowerment of kindergarten teachers in minority areas. The education administrator should maintain and strengthen the levels of teachers' psychological empowerment based on the four aspects of meaning, competence, self-determination, and impact. Specifically, kindergarten directors could appropriately grant teachers increased autonomy, which could boost teachers' competence and enable them to quickly assume responsibility and resolve unanticipated challenges that may occur during kindergarten teaching in ethnic areas (Lee and Nie, 2013, 2014). Furthermore, kindergarten leaders should also communicate with teachers about the kindergarten's vision, values, and policies and discuss kindergarten plans or decisions, which could promote kindergarten teachers to realign their personal values with organizational goals (Chan et al., 2008; Lee and Nie, 2014), thereby increasing employees' sense of meaning at work. By doing so, it will help to strengthen the construction of teachers in less developed areas, improve the overall quality of education in a region or country, and thus benefit more developing children and adolescents.

## Conclusion

Teachers' retention is an important dilemma faced by kindergarten in ethnic minority areas in China. This study investigated the underlying mechanisms accounting for the associations between kindergarten climate and retention intention. Kindergarten climate is a set of measurable attributes perceived directly or indirectly by employees in their work conditions, which could influence individual motivation and behavior in that work condition. This study suggested that kindergarten climate could positively predict retention intention through a separate indirect path *via* perceived organizational support and psychological empowerment. Meanwhile, kindergarten climate may also be associated with retention intention through the chain mediating effect of perceived organizational support and psychological empowerment. These findings provide a theoretical foundation for improving teachers' retention intention, as well as some practical guidance for local education departments and kindergarten administrators in developing effective interventions for promoting teachers' retention intention in ethnic minority areas of China.

## Data availability statement

The data presented in this study are available on request from the corresponding authors.

## Ethics statement

The studies involving human participants were reviewed and approved by Ethics Committee of Minzu University of China. The participants provided their written informed consent to participate in this study.

## Author contributions

DS and MZ planned the design of the study, organized the data collection, and drafted the Methods section. DS, MZ, and XY drafted the Introduction, Results, and Discussion sections, contributed to an adequate statistical implementation of the presented idea, and computed the statistical analyses. DS, MZ, XY, YX, and YW revised and perfected the thesis. All authors contributed to the manuscript and approved the submitted version.

## Funding

This research was supported by the Graduate Research and Practice Projects of Minzu University of China (GTTZX2022009) and Ethnic Research Project of the National Ethnic Affairs Commission of the People's Republic of China in 2021 (2021-GMI-010).

## Conflict of interest

The authors declare that the research was conducted in the absence of any commercial or financial relationships that could be construed as a potential conflict of interest. The reviewer J-HY declared a shared affiliation, with no collaboration, with one of the authors XY, to the handling editor at the time of the review.

## Publisher's note

All claims expressed in this article are solely those of the authors and do not necessarily represent those of their affiliated organizations, or those of the publisher, the editors and the reviewers. Any product that may be evaluated in this article, or claim that may be made by its manufacturer, is not guaranteed or endorsed by the publisher.
