# Supplementary material for: Associations between kindergarten climate and retention intention of kindergarten teachers: The chain mediating roles of perceived organizational support and psychological empowerment
Source: Front Psychol. 2022 Aug 1;13:906434. doi: 10.3389/fpsyg.2022.906434 (PMC9377455; doi:10.3389/fpsyg.2022.906434)
Supplement: Supplementary file 1 [file Table_1.docx]

**SUPPLEMENT MATERIAL 1 |** Demographic information.

| **Variables** | **Types** | **Number** | **Percent %** |
| --- | --- | --- | --- |
| Gender | Male | 40 | 3.3 |
|  | Female | 1159 | 96.7 |
| Age | 20 and below | 71 | 5.9 |
|  | 21-25 | 413 | 34.4 |
|  | 26-30 | 295 | 24.6 |
|  | 31-35 | 162 | 13.5 |
|  | 36-40 | 105 | 8.8 |
|  | 41-45 | 68 | 5.7 |
|  | 46-50 | 64 | 5.3 |
|  | 51 and above | 21 | 1.8 |
| Ethnic | Han | 768 | 64.1 |
|  | Hui | 402 | 33.5 |
|  | Other minorities  (Bai, Tibet, Dai, Lisu, Man, Miao, Yi) | 29 | 0.24 |
| Educational level | Primary school and below | 7 | 0.6 |
|  | junior high school | 29 | 2.4 |
|  | Senior high school/technical secondary school | 247 | 20.6 |
|  | Bachelor | 911 | 76 |
|  | Master and above | 5 | 0.4 |
| Seniority | Novice teachers | 878 | 73.2 |
|  | Veteran teachers | 321 | 26.8 |
