# Supplementary material for: Associations between kindergarten climate and retention intention of kindergarten teachers: The chain mediating roles of perceived organizational support and psychological empowerment
Source: Front Psychol. 2022 Aug 1;13:906434. doi: 10.3389/fpsyg.2022.906434 (PMC9377455; doi:10.3389/fpsyg.2022.906434)
Supplement: Supplementary file 2 [file Table_2.docx]

**SUPPLEMENT MATERIAL 2 |** Regression analysis of the relationship between kindergarten climate and retention intention.

| Result variable | Predictor variable | Significance | | |  | Fitting index | | |
| --- | --- | --- | --- | --- | --- | --- | --- | --- |
|  |  | *β* | *SE* | *t* |  | *R* | *R²* | *F* |
| Retention intention |  |  |  |  |  | 0.493 | 0.243 | 96.06^***^ |
|  | Seniority | -0.062 | 0.072 | -0.865 |  |  |  |  |
|  | Age | 0.061 | 0.019 | 3.193 |  |  |  |  |
|  | gender | 0.054 | 0.148 | 0.363 |  |  |  |  |
|  | kindergarten climate | 0.466 | 0.024 | 19.219^***^ |  |  |  |  |
